# Cardiac compression by a large pericardial cyst in hydatidosis

**DOI:** 10.1093/jscr/rjag397

**Published:** 2026-06-22

**Authors:** Bobur Ataev, Balhen Bolathan, Weimin Zhang

**Affiliations:** Department of Cardiac Surgery, The First Affiliated Hospital of Xinjiang Medical University, No. 137 Liyushan RD, Urumqi, Xinjiang 830011, China; Khiva District Station, Khorezm Branch of the Republican Ambulance Center of Uzbekistan, 1 K. Otaniyozov Street, Urgench, Khorezm region 220500, Uzbekistan; Department of Cardiac Surgery, The First Affiliated Hospital of Xinjiang Medical University, No. 137 Liyushan RD, Urumqi, Xinjiang 830011, China; Department of Cardiac Surgery, The First Affiliated Hospital of Xinjiang Medical University, No. 137 Liyushan RD, Urumqi, Xinjiang 830011, China

**Keywords:** pericardial hydatidosis, echinococcosis, pericardium, transesophageal echocardiography, hypertonic saline, albendazole

## Abstract

Pericardial hydatidosis is exceptionally rare. However, it can lead to cardiac tamponade or anaphylaxis. A 36-year-old farmer presented with chest tightness and dyspnea. Multimodality imaging illustrated a large, heterogeneous, avascular cystic mass within the pericardium adjacent to the right atrium and ventricle, causing significant compression and deformation of these chambers. Elective surgery was performed under transesophageal echocardiographic guidance to ensure precise resection of the cardiac echinococcal lesion and effective reduction of residual pathology. The postoperative course was favorable. A high index of suspicion is required for patients with a pericardial mass who originate from endemic regions or have a history of animal contact. The size and location of the cyst and the presence of complications determine the clinical presentation. Diagnosis is mainly based on cardiac imaging techniques. Surgical excision is the best treatment method for pericardial cystic echinococcosis. Intraoperative transesophageal echocardiography may serve as a valuable adjunct tool.

## Introduction

Cardiac involvement is rare in cystic echinococcosis, occurring in only 0.5%–2% of cases [1]. Within this already uncommon group, pericardial involvement is even rarer, and is found in just 8% of cardiac hydatid cases [2]. The size and location of the cyst, its compressive effects on adjacent structures, and the presence of complications determine the clinical presentation [1, 3]. Chest pain, compression of coronary vessels, dyspnea, and palpitations are the most frequent symptoms of uncomplicated pericardial cystic echinococcosis. Pericardial hydatidosis can remain asymptomatic or may lead to even lethal complications such as cardiac tamponade and anaphylactic shock [4]. Cyst perforation occurs in 25%–40% of cases, and it represents the most serious complication of pericardial hydatid disease [5].

## Case presentation

A 36-year-old female farmer from Xinjiang, an *Echinococcus*-endemic region [2], presented to our hospital with chief complaints of chest tightness and dyspnea that had persisted for the last 5 days. The symptoms began without an obvious cause, lasted for ~3 min per episode, and were relieved by rest. She also had a cough, sputum production, and dizziness. The patient had undergone a previous hepatic hydatid cyst resection 8 years ago.

On her physical examination, a systolic murmur was heard at the apex of the heart. Transthoracic echocardiography illustrated an 11.1 × 6.6 × 5.5 cm, heterogeneous, avascular cystic mass within the pericardium adjacent to the right atrium and the right ventricle, causing significant compression and deformation of these chambers (Fig. 1A). Contrast-enhanced echocardiography confirmed its avascular, cystic nature (Fig. 1B). Chest computed tomography (CT) confirmed the presence of a cystic mass within the pericardium, adjacent to the right cardiac margin (Fig. 2A). It demonstrated ill-defined borders with the walls of the right atrium and ventricle, causing significant compression of these chambers (Fig. 2B). Cardiac magnetic resonance imaging (MRI) further confirmed a large heterogeneous cystic mass within the right pericardial cavity, characterized by mixed T1- and T2-weighted signal intensities and heterogeneous enhancement, resulting in significant compression of the right atrium and ventricle without evidence of myocardial invasion (Fig. 2C). The lesion exhibited characteristic features of a complex cyst and the presence of internal daughter cysts (Fig. 2C and D).

**Figure 1:**
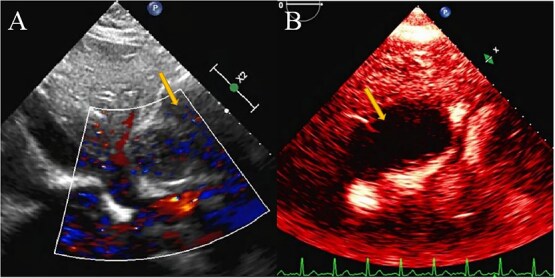
Pericardial hydatidosis on echocardiography. (A) Transesophageal echocardiography showed a large compressive pericardial mass. (B) Contrast-enhanced echocardiography confirmed its avascular, cystic nature. Arrow: pericardial hydatid cyst.

**Figure 2:**
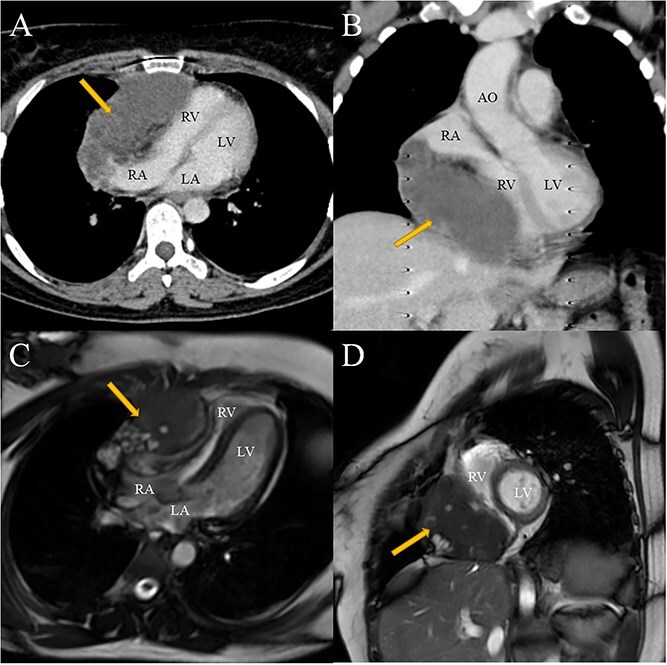
CT and MRI data. (A, B) Contrast-enhanced chest CT revealed a well-defined, oval-shaped cystic mass within the pericardial space adjacent to the right cardiac border, leading to compression of the right atrium and right ventricle. (C, D) Cardiac MRI demonstrated a well-circumscribed mass in the right pericardial cavity with heterogeneous signal intensity, resulting in compression of the right atrium and right ventricle. The lesion displayed characteristic features of a complex cyst, including the presence of internal daughter cysts. Arrow: pericardial hydatid cyst. AO, aorta; LA, left atrium; LV, left ventricle; RA, right atrium; RV, right ventricle.

The patient was started on oral albendazole (200 mg twice a day) 1 week prior to surgery. The patient underwent a planned surgical resection of the pericardial hydatid cyst and partial pericardiectomy on a beating heart through a median sternotomy under general anesthesia. The pathological mass was firmly adherent to the right atrium and ventricle. Initially, the remaining part of the pericardium was isolated using gauze pads soaked in a 20% saline solution (Fig. 3A). Subsequently, a cystotomy was performed to evacuate the hydatid fluid and remove the endocyst, daughter cysts, and necrotic debris (Fig. 3B and D). Partial excision of the pericyst was carried out following irrigation of the cyst cavity with 20% hypertonic saline (Fig. 3C). The surgical intervention was performed under esophageal ultrasound guidance to ensure precise resection of the cardiac echinococcal lesion and effective reduction of residual pathology.

**Figure 3:**
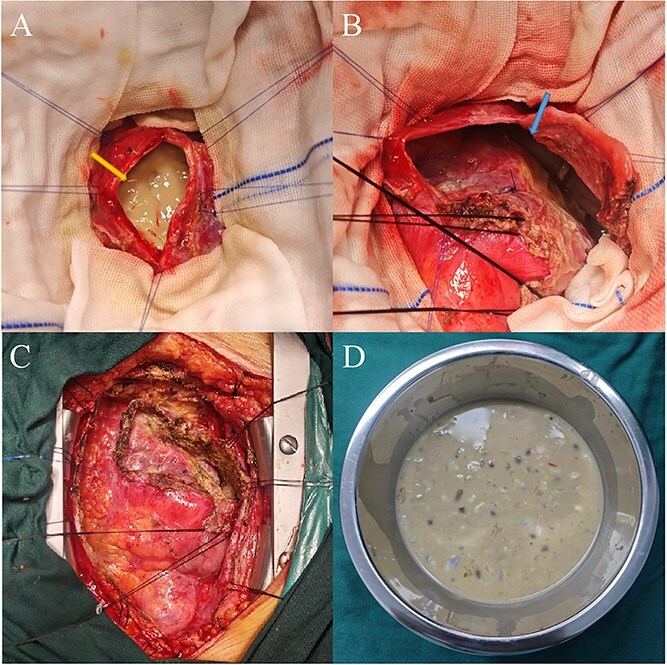
Intraoperative findings and surgical procedure for pericardial cystic echinococcosis. (A) The hydatid cyst (yellow arrow) is located within the pericardium, adjacent to the right cardiac border. (B) The residual cavity (blue arrow) after complete aspiration of hydatid fluid and removal of daughter cysts and necrotic tissues. (C) Intraoperative view following partial excision of the pericyst. (D) Aspirated cyst fluid, necrotic tissue, resected endocyst, and daughter cysts. Yellow arrow: pericardial hydatid cyst; blue arrow: postoperative residual cavity.

Cytology of the aspirated cyst fluid and histology of the cyst wall were consistent with the hydatid nature of the cyst (Fig. 4). The postoperative course was favorable. Albendazole therapy was continued postoperatively for 12 weeks. The patient was discharged in good condition on the ninth postoperative day. She remained asymptomatic and showed no evidence of recurrence at the routine follow-up examination 6 months postoperatively (Fig. 5).

**Figure 4:**
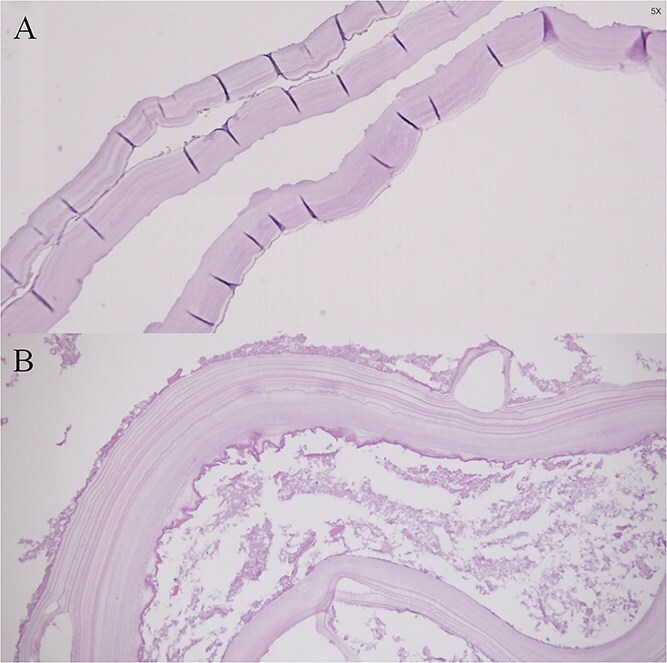
Histopathological findings demonstrating the laminated membrane and germinal layer of the hydatid cyst wall.

**Figure 5:**
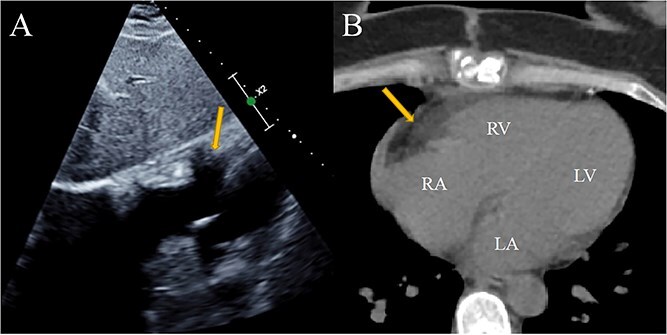
Six-month postoperative imaging follow-up after surgical resection of pericardial hydatid cyst. (A) Echocardiography confirming complete resection of the pericardial hydatid cyst with no evidence of residual or recurrent pathology. (B) Chest CT demonstrating normal cardiac anatomy without residual cystic lesion or compression of the cardiac chambers. Arrow: previous location of the pericardial hydatid cyst. LA, left atrium; LV, left ventricle; RA, right atrium; RV, right ventricle.

## Discussion

Early diagnosis and urgent treatment are crucial to prevent complications [3]. Cardiac imaging techniques are the basis of the diagnosis of pericardial hydatid disease [5]. Transthoracic echocardiography is a common, safe, and easy method for diagnosis, and its sensitivity is high in the detection of pericardial masses and surgical planning [3, 5, 6]. Although cardiac hydatidosis is rare, routine transthoracic echocardiography screening may be recommended for all individuals diagnosed with cystic echinococcosis [5]. CT and MRI are effective to confirm diagnosis, define the location and size of the cyst and its adhesions and relationship to adjacent structures, and look for lesions in other locations [2, 3]. CT is the best imaging modality for illustrating wall calcification [5]. MRI provides the best visualization of the anatomical proximity of cystic echinococcosis, the state of the cyst structure, and the precise anatomic location [5, 7].

Surgical excision is the best treatment method for pericardial cystic echinococcosis due to the risk of progressive and potentially fatal complications in its natural course, even in asymptomatic patients [2, 5, 8]. The postoperative recurrence rate of all hydatid diseases is approximately 10% [2]. Therefore, intraoperative preventive measures, such as the use of hypertonic saline as a scolicidal agent, are essential to avoid dissemination and recurrence [3]. With regard to the use of transesophageal echocardiography during the surgical management of cardiac hydatid disease, direct clinical guidelines or published studies that provide conclusive evidence supporting its routine use are currently lacking. The patient presented with an exceptionally large pericardial hydatid cyst involving an extensive anatomical region. Intraoperative transesophageal echocardiography was employed to guide the surgical intervention, ensuring precise lesion localization and safe excision. Postoperative evaluation by transthoracic echocardiography and chest CT confirmed the absence of residual disease (Fig. 5). In instances of multiple and intricate pericardial hydatid cysts, the extensive involvement of affected tissues presents substantial challenges to surgical exploration and exposure, frequently making complete resection unattainable. The intraoperative application of transesophageal echocardiography allows for accurate localization of lesions, enhances surgical planning and execution, reduces the likelihood of residual disease, and consequently contributes to a decrease in postoperative recurrence rates.

## Conclusion

A high index of suspicion is required for patients with a pericardial mass who originate from endemic regions or have a history of animal contact. Timely diagnosis and appropriate intervention help prevent the severe complications of disease progression. Diagnosis is mainly based on cardiac imaging techniques. Surgical excision remains the cornerstone of treatment for pericardial hydatid disease. In selected complex cases, intraoperative transesophageal echocardiography may serve as a valuable adjunct by facilitating accurate lesion localization, guiding surgical excision, and minimizing the risk of residual disease.

## Data Availability

The images and data used and/or analyzed during the current study are available from the corresponding author upon reasonable request.
